# Modified Cellulose Proton-Exchange Membranes for Direct Methanol Fuel Cells

**DOI:** 10.3390/polym15030659

**Published:** 2023-01-27

**Authors:** Gowthami Palanisamy, Tae Hwan Oh, Sadhasivam Thangarasu

**Affiliations:** School of Chemical Engineering, Yeungnam University, Gyeongsan 38541, Republic of Korea

**Keywords:** fuel cell, cellulose membrane, Nafion membrane, hydrocarbon membrane, proton-exchange membrane, direct methanol fuel cell, methanol permeability, cost-effective, high performance, proton conductivity

## Abstract

A direct methanol fuel cell (DMFC) is an excellent energy device in which direct conversion of methanol to energy occurs, resulting in a high energy conversion rate. For DMFCs, fluoropolymer copolymers are considered excellent proton-exchange membranes (PEMs). However, the high cost and high methanol permeability of commercial membranes are major obstacles to overcome in achieving higher performance in DMFCs. Novel developments have focused on various reliable materials to decrease costs and enhance DMFC performance. From this perspective, cellulose-based materials have been effectively considered as polymers and additives with multiple concepts to develop PEMs for DMFCs. In this review, we have extensively discussed the advances and utilization of cost-effective cellulose materials (microcrystalline cellulose, nanocrystalline cellulose, cellulose whiskers, cellulose nanofibers, and cellulose acetate) as PEMs for DMFCs. By adding cellulose or cellulose derivatives alone or into the PEM matrix, the performance of DMFCs is attained progressively. To understand the impact of different structures and compositions of cellulose-containing PEMs, they have been classified as functionalized cellulose, grafted cellulose, acid-doped cellulose, cellulose blended with different polymers, and composites with inorganic additives.

## 1. Introduction

Over recent decades, significant research has been devoted to the generation of energy from renewable energy sources to avoid environmental issues related to fossil fuel extraction and utilization [1,2]. However, direct utilization of renewable energy sources, such as solar and wind energy, is not feasible in many cases because of the issues of instability and intermittency during generation, which complicates their stable and continuous application [3]. Thus, different types of energy storage and generator systems have been developed to efficiently utilize energy carriers derived from renewable energy sources [4,5,6]. Fuel cell-based technologies have received significant attention due to their good reliability, high energy density, environmental friendliness, and safety, which are significant concerns for the ecosystem and human life [7,8,9,10,11,12,13,14,15]. Relying on fuel cell technologies, various developments and approaches have focused on attaining the greatest benefits during practical applications. Progress has been made in addressing the challenges to fuel cell efficiency by considering different operating conditions, utilizing different fuels and components [16,17]. Accordingly, various fuel cells have been termed, such as low- [18], intermediate- [19], and high-temperature [20] proton-exchange membrane fuel cells (PEMFCs) [21,22], alkaline fuel cells [23,24,25,26], direct methanol fuel cells (DMFCs) [27,28], direct ethanol fuel cells [29], molten-carbonate fuel cells [30], direct borohydride fuel cells [31], solid-oxide fuel cells [32], unitized-regenerative fuel cells [33,34], and microbial fuel cells [35]. In recent years, considerable attention has been paid to DMFCs [36,37,38,39,40]. Because of its convenient storage, easy transference, production with sustainable biomass resources or natural gas, low cost, and high volumetric energy density, methanol is preferable in fuel cell technologies [27,28,41,42,43,44,45,46]. DMFCs have been considered because of their numerous benefits, such as ease of operation, efficient performance in low-temperature operation, high specific energy, easy refilling of fuel, safety, higher efficiency, and low environmental pollution [27,28,42,43]. DMFCs are considered efficient energy devices for various applications, such as stationary power plants, electronic vehicles, unmanned aerial vehicle power, portable electronic devices, forklift power, single soldier power, and backup power for laptops [27,42,47,48]. At the anode, the methanol oxidation reaction (MOR) occurs, where the combination of methanol and water generates six electrons and protons with a byproduct of carbon dioxide (CO_2_) [49,50,51,52]. To complete the reaction, electrons and protons move to the cathode side through an external electric circuit and proton transport membrane. The oxygen reduction reaction (ORR) occurs in the cathode electrocatalyst by reacting protons, electrons, and oxygen, where water molecules exist as a byproduct [53,54,55,56]. DMFCs release greenhouse gas emissions (CO_2_) during unit cell operation. DMFCs may be utilized as a low-carbon emission alternative, even if they are not as environmentally friendly as PEMFCs that utilize green hydrogen.

The performances of most energy conversion and storage devices are related to the properties of the electrode and membrane materials [41,57,58,59,60]. Therefore, the most important part is the membrane electrode assembly (MEA), which contains a proton-exchange membrane (PEM) and electrodes [27,61,62]. The polymer membrane separates the anode and cathode. The significant role of the PEM in MEA is to transport protons from the anode to the cathode, prohibiting methanol crossover and preventing short-circuit-related issues. PEMs are considered as an efficient PEM for DMFCs due to their high proton conductivity, lower methanol permeability and oxygen, easier availability, low cost, high chemical stability, efficient electrochemical steadiness, and higher mechanical and thermal stability [63,64,65]. The commercial polymer membranes (such as DuPont (Nafion^®^), Asahi Glass Engineering (FlemionR^®^), Fumatech (Fumion^®^), Solvay (Aquivion^®^), Asahi Kasei (Aciplex-S^®^), and Dow Chemicals (XUS^®^)) have been effectively considered as PEMs [63,65,66,67,68]. Perfluorosulfonic acid (PFSA) contains two hydrophobic and hydrophilic phases, where the polymer backbone is the hydrophobic phase and the side chain containing the sulfonic acid functional group is the hydrophilic phase [69,70,71,72]. Owing to the existence of two phases in the PFSA membrane, PFSA-based membranes provide excellent properties, such as higher proton conductivity and excellent mechanical properties [69,73,74,75,76]. PFSA-based membranes are associated with certain disadvantages, such as (i) high production cost, (ii) high methanol crossover, (iii) lower proton conductivity at low humidity, (iv) unsteady proton conductivity at high temperatures due to dehydration, and (v) the possibility of environment-related issues, where the attack of hydroxyl (•OH) radicals induces the loss of fluorine species from the membrane [63,64,65]. To overcome these issues, numerous efforts and developments have been made to expand and boost the probability of DMFC commercialization [63,64,77,78,79,80]. The developments focused on replacing or decreasing the cost and increasing the selectivity of commercial membranes in two ways: (i) including the different characteristic properties of polymer materials or inorganic nanofillers in the PFSA polymer matrix without significantly affecting the overall performances [81,82,83,84,85,86], and (ii) searching for alternative polymer membranes with and without the addition of functional groups, polymers, inorganic fillers, and/or cross-linkers as a blend, composite, or cross-linked membranes [87,88,89,90,91,92,93,94]. There are different kinds of synthetic polymers, such as sulfonated poly(ether ether ketone) [91,95,96], sulfonated polysulfone [97], sulfonated poly(vinylidene fluoride) [98], polytetrafluoroethylene [99], and poly(phenylene oxide) [100] that have been studied to improve the performances of DMFC. In addition, different blend membranes, composite membranes, grafted membranes, and cross-linked membranes have been established for DMFC. Moreover, natural and semi-synthetic polymer membranes have been effectively considered for DMFC applications in recent years.

Bio-based polymers (natural and semi-synthetic) with or without ion-exchange properties have been effectively considered as membrane and separator materials for various energy storage and conversion systems, such as fuel cells, batteries, and supercapacitors [101,102,103,104,105,106,107,108]. Biopolymers, such as cellulose, chitosan, lignin, and alginate, possess numerous benefits for energy systems, primarily in terms of abundance and cheaper source materials [109,110,111,112]. Cellulose has been widely considered as a resourceful membrane material for different energy systems, including DMFC. Cellulose possesses numerous valuable properties such as low production cost, high purity, plenty of hydroxyl functional groups, good hydrophilic properties, reasonable water uptake capability, waste-to-value-added products, biodegradability, renewability, environmental friendliness, and, most importantly, the possibility of functionalization of other functional groups, mechanical properties, and compatibility [113,114,115,116,117,118]. Commonly, cellulose is derived from various sources, such as plants and bacteria. Based on the source, structural properties, size, and functional properties, cellulose materials are classified into different categories, such as bacterial nanocellulose (BNC), cellulose nanocrystals (CNCs), cellulose microcrystals (CMCs), cellulose whiskers (CWs), cellulose nanofibers (CNFs), and cellulose acetate (CA). This review focuses on recent progress in modified cellulose-based composite membranes as PEM for DMFC operations. Different forms of cellulosic materials (CMC, CNC, CW, CNF, and CA) and their impact are also presented. Membrane modification in terms of cross-linking, grafting, composite, and blend inorganic material incorporation influences membrane behavior and alters physicochemical properties. The impact of membrane stability, lowering methanol permeability, and DMFC performance of cellulose materials are reviewed in detail.

## 2. Microcrystalline, Nanocrystalline, and Nanowhisker Cellulose-Containing PEMs for DMFCs

**Figure 1:**
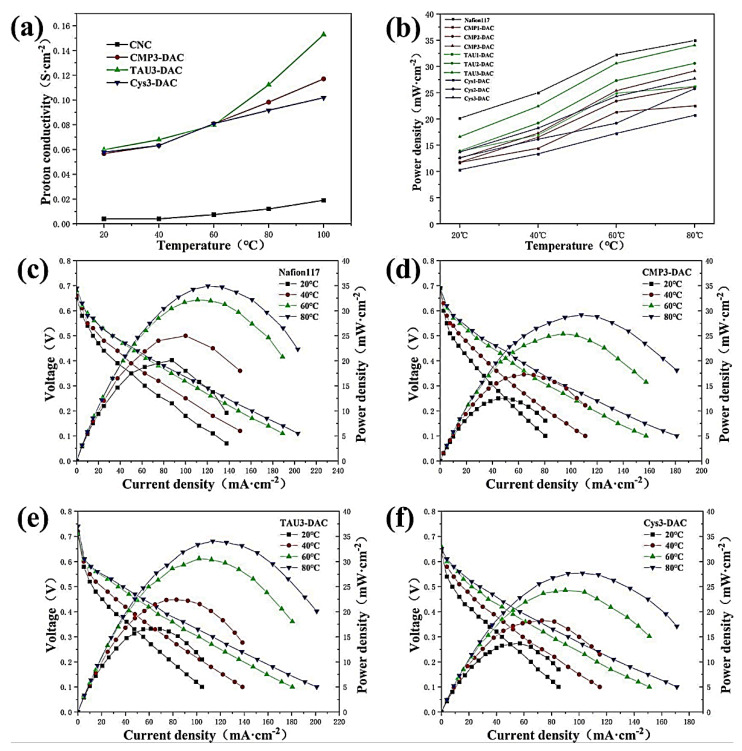
(**a**) Proton conductivities; (**b**) power densities of Nafion, CNC, and modified CNC membranes. DMFC polarization and power density curves of (**c**) Nafion 117, (**d**,**e**) CNC grafted with CMP–DAC and TAU–DAC, respectively, and (**f**) Cys–DAC membranes. Reprinted with permission from Ref. [130]. Copyright © 2022, American Chemical Society.

Microcrystalline cellulose (MCC) is obtained by partially hydrolyzing the amorphous region of cellulose. Crystalline and purified MCC can be obtained using different methods, such as acid hydrolysis, alkali hydrolysis, steam explosion, extrusion, or the radiation-enzymatic process [119,120,121]. MCC usually measures 50–500 μm in diameter, with a length of more than 1 μm [122,123]. To reduce manufacturing costs, MCC is further processed to obtain CNC. CNCs have the potential to be used as nanomaterials to make inexpensive, lightweight, and robust nanocomposites [124,125,126]. High Young’s modulus and tensile strength are features of CNC with nanosized diameters of 1–100 nm and lengths of 10–1000 nm [124]. MCC and CNC possess significant properties such as a high aspect ratio, large surface area, high water uptake, biodegradability, biocompatibility, and enhanced mechanical and barrier properties [127,128,129]. Therefore, MCC and CNC have been used to develop PEM for fuel cell applications. Huang et al. grafted CNCs with biomass-derived cytidine monophosphate (CMP), taurine (TAU), and cysteine (Cys) to improve their proton conductivity for DMFC applications [130]. During grafting, CNCs are first transformed into 2,3-dialdehyde cellulose (DAC) by the action of an oxidant (NaIO_4_). Then, acetic acid functioned as a catalyst to enable nucleophilic primary aldehyde groups on DAC to interact with the changed molecules in a Schiff-base process [130,131,132]. Hemiacetal may be generated by the influence of a possible cross-linking between the hydroxyl (−OH) on CNCs and the aldehyde groups (CHO) on DAC, which augments the tensile characteristics of the membrane [103,130]. Additionally, the tensile strength can be improved by the interfacial interaction between the CNC and polymer. The modified membranes were designated as CMP-DAC, TAU-DAC, and Cys-DAC. A reasonable number of sulfonic acid groups in TAU3-DAC attributed to higher proton conductivity of 0.1528 Scm^−1^ at 100 °C (Figure 1a). The sulfonic and phosphoric groups in the membrane served as the proton donors and acceptors, respectively. The proton transfer distance was shortened by the addition of CMP, TAU, and Cys to the membrane. The decreased substitutional degree and exchangeable proton capacity of the modified membranes resulted in a lower ion-exchange capacity (IEC) value (between 0.0344 and 0.3267 mmol g^−1^) than that of the Nafion117 membrane (between 0.345 and 0.95 mmol g^−1^) at 20 and 80 °C, respectively [130]. Figure 1b–f show the power density and polarization curves of the Nafion117 and modified membranes. In contrast to the pure CNC membrane, which had no power output, the Nafion117 membrane had a power output of 34.95 mW/cm^2^ at 80 °C. The power density of CNC-containing membranes was increased by modifying the concept. Meanwhile, the existence of phosphate or sulfonate groups in the membranes is responsible for the abundance of proton transport sites. The highest power density was measured for the TAU3-DAC membrane (34.05 mW/cm^2^). The obtained power density of the other TAU3-DAC membrane was approximately 97.4% of the Nafion117 membrane performance [130]. In another approach, Zhao et al. developed a new proton conductive membrane with 2,6-diaminopurine grafted onto CNC [133]. At high temperatures (100 °C), the modified CNC membrane exhibited higher proton conductivity of up to 0.222 S cm^−1^, which was greater than that of the pure CNC membrane (0.019 Scm^−1^). Moreover, the modified CNC membrane demonstrated an increased tensile strength of 91.35 MPa, suggesting greater mechanical strength and flexibility. Compared to the commercial membrane (2.09 × 10^−6^ cm^2^/s), the composite membranes showed a much lower methanol permeability (1.41 × 10^−7^ cm^2^/s) and efficient thermal stability [133].

**Figure 2:**
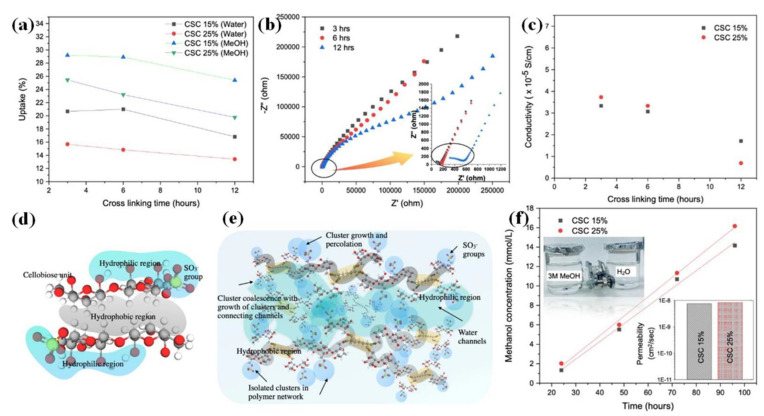
Cross-linked sulfated cellulose membranes. (**a**) Water and methanol uptake. (**b**) Nyquist plot of the 25% GA cross-linked membrane. (**c**) Proton conductivity. (**d**) Formation and (**e**) cluster-network mode of cross-linked membranes. (**f**) Methanol crossover and permeability of cross-linked sulfated cellulose membranes [134].

The cross-linking of sulfated cellulose (SC) membranes by a combination of SC from acid hydrolysis MCC and Glutaraldehyde (GA) showed considerable performance improvement in DMFC applications [134]. An increase in the hydrophobic backbone domains and cross-linking confirmation was attributed to the connection between the GA aldehyde groups and cellulose primary −OH groups. As indicated in Figure 2a, the water uptake and methanol uptake capacity were effectively influenced in cross-linked sulfated cellulose membranes with respect to cross-linking time (3, 6, and 12 h) and GA amount. It was observed (Figure 2b,c) that the conductivity decreased in cross-linked sulfated cellulose (CSC) membrane with 15% GA from 3.3 × 10^−5^ S/cm to 1.7 × 10^−5^ S/cm at 25 °C, whereas the reaction time increased from 3 h to 12 h. Similarly, a three-fold decrease in conductivity was observed for membranes with 25% GA (3.7 × 10^−5^ S/cm to 0.69 × 10^−5^ S/cm). This was due to the increased hydrophobic region during cross-linking, which reduced the water uptake responsible for proton transfer. Additionally, the decrease in proton conductivity possibly occurred because of a decrease in free volume caused by cross-linking behavior, which lowered the proton mobility in the water channels and reduced proton conductivity. Furthermore, the cross-linked network probably prevents the formation of efficient ionic clusters, which may also weaken ionic conductivity. This phenomenon reveals that the proton conductivity of the cross-linked sulfated cellulose membranes is altered by the degree of cross-linking in the membrane matrix. Moreover, the GA concentration and short cross-linking time (3 and 6 h) also impacted the conductivity of the membranes. Among the different concepts, shorter (3 h) reaction times with higher GA concentrations (25%) provided the best conditions for producing a CSC membrane with high performance. Consequently, stacking by hydrophobic interactions may lead to a sheet-like structure (Figure 2d) [134,135]. Moreover, the hydrophilic interaction of cellulose is enhanced by the presence of the −SO_3_^−^ functional group in sulfated cellulose. Owing to the hydrophilic (−SO_3_^−^ functional group) and hydrophobic (cellulose backbone) properties of CSC, the phase separation is strengthened during solvation. Similar to the cluster-network concept in perfluorinated polymers [134,136,137], CSC with increased hydrophilicity and hydrophobicity provides a capable water channel in the membrane (Figure 2e). As shown in Figure 2f, methanol permeation through the CSC membranes was measured in a two-compartment H-type glass diffusion cell, and the corresponding results are represented as the variation in methanol concentration in the water compartment versus time and methanol permeability. Compared with the CSC membrane made with 15% GA, the CSC membrane made with 25% GA provided more excellent resistance to methanol permeability. The methanol permeability of the 25% GA of CSC revealed 8.28 × 10^−9^ cm^2^/s at 3M [134].

In the development of PEM using biopolymers, the incorporation of inorganic materials has been a significant consideration for enhancing the stability in terms of mechanical, oxidative, thermal, and dimensional properties [138]. Furthermore, the presence of inorganic materials in the membrane matrix probably alters the water-upholding behavior, IEC, and proton conductivity. As an example, phosphotungstic acid (PTA), a type of heteropoly acid (HPA), possesses high acidity and stability [139,140]. A high power density, which is excellent for fuel cell applications, may be produced by the high tungsten oxidation level and the reduction of W^6+^ to W^3+^. Additionally, imidazole (Im) (a heterocyclic organic material) possesses a lone pair of electrons that aids in maintaining the proton transfer mechanisms [140]. MCC treated with Im and PTA through a phase inversion approach improved the performance of MCC for DMFCs [141]. The intramolecular hydrogen bond was not altered by PTA, as revealed by the IR spectra peaks around 900 and 1420 cm^−1^ for glycosidic and CH_2_ vibrations, respectively. The modified cellulose membranes provided enhanced proton conductivity compared to that of the unmodified cellulose membranes, where the proton conductivities were 0.073, 0.106, and 0.214 mS/cm for cellulose, PTA-cellulose, and Im-cellulose, respectively. The results show that modifying MCC with Im and PTA may reduce methanol permeability while improving the water management of the modified membranes [141]. In another study, a pristine nanocellulose (NC) membrane was also incorporated with 2 wt% PTA and 5 wt% Im to develop a composite membrane using a similar phase inversion technique [139]. In this case, the cellulose structure changed from microparticles to ~88.79 nm in size. It was observed that the NC/Im membrane had the best proton conductivity compared to that of the other membranes. The obtained proton conductivity is 5.32, 6.34, 13.17, and 14.98 mS/cm for the NC, NC-Im-PTA, NC-PTA, and NC-Im membranes, respectively. The inclusion of Im and PTA in the NC membranes altered the IEC, which was the primary reason for the higher proton conductivity. Further, the NC/Im membrane exhibited lower methanol permeability than Nafion. Thus, the NC/Im membrane attained the most significant selectivity (2 × 10^4^ S.s/cm^3^) among all the membranes [139]. Recently, Priyangga et al. reported the development of ternary membranes combining NC, Im, and mesoporous phosphotungstic acid (m-PTA) [140]. To avoid the easy dissolution of PTA in the solvent, PTA was modified into the mesoporous form. The self-assembly approach was used to effectively develop m-PTA fillers with a pore size of 4.89 nm. Additionally, the membrane contained NC-Im-m-PTA-5, which demonstrated the most remarkable results regarding its water uptake (50.68%), IEC (1.885 mmol g^−1^), proton conductivity (31.88 mS/cm) and selectivity (1.83 × 10^4^ S/cm^3^). Additionally, the NC-Im-m-PTA-5 membrane controls the methanol permeability (1.74 × 106 cm^2^/s) and methanol uptake (3.19%) [140].

**Figure 3:**
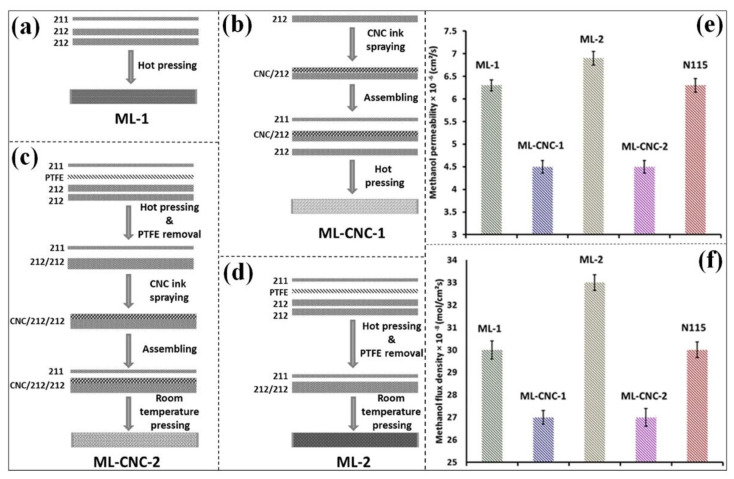
Schematic representation of different multi-layer (ML) membranes preparation processes: (**a**) ML–1 without CNC layer, (**b**) ML–CNC–1 with CNC layer, (**c**) ML–CNC–2 with CNC layer and room temperature pressing, and (**d**) ML–2 without CNC layer and room temperature pressing. (**e**) Methanol permeability; (**f**) methanol flux density of membranes at 70 °C [145].

The modification of commercial PEMs (e.g., Nafion membranes) and composites with commercial PFSA membranes provides significant enhancement in performance as well as a decrease in the overall membrane cost [142,143,144]. To identify the impact of CNCs on commercial membranes, a multi-layer (ML) membrane comprising Nafion 211 and Nafion 212 with a spray-coated CNC layer for DMFCs was prepared (Figure 3) [145]. As shown in Figure 3a–d, the ML membranes were developed with and without the CNC layer (ML-1, ML-2, ML-CNC-1, and ML-CNC-2) by the hot press method to evaluate the impact of the presence of CNCs in the compressed ML membranes. As shown in Figure 3e, the methanol crossover and permeability of the CNC layer-compressed membrane were considerably lower than those of the ML-1, ML-2, and Nafion 115 (N115) membranes. The methanol flux densities for all membranes (Figure 3f) characterize the actual quantity of methanol crossing the membrane. The methanol flux density of ML-1 was similar to that of the N115 membrane. The methanol flux densities of the ML-1 and N115 membranes were ~30 × 10^−8^ mol/cm^2^s. However, ML-2 had a methanol flux density that was 10% greater than that of the N115 membrane. These results suggest that the individual layers of ML-2 had less mechanical bonding, which allowed methanol to cross through the membrane. This was supported by the water uptake measurements. ML-CNC-1 and ML-CNC-2 exhibited the lowest methanol flux densities compared to that of the pristine ML-1 and N115 membranes. This was primarily due to the presence of the CNC layer in the multi-layer membranes, which was attributed to the crystallinity and barrier characteristics of the CNCs. As compared to the ML-CNC-1 membrane, the ML-CNC-2 attained a higher proton conductivity at different methanol concentrations (1, 2, and 4 M) at 70 °C. Because of the removal of functional groups from the CNC surface during heat treatment, ML-CNC-1 (hot-pressed CNC) had a lower proton conductivity value than ML-CNC-2. The incorporation of CNCs in ML membranes reduced the proton conductivity compared to that of commercial membranes but significantly enhanced the methanol barrier. Thus, ML-CNC attained a higher selectivity, which provided a significant enhancement in the DMFC unit cell performance. Among the different ML membranes and the N115 membrane, the most significant performance over the whole range of methanol concentrations studied was attained with room-temperature-pressed CNCs (ML-CNC-2). These results indicate that the low concentrations (1.5% in the composite membrane) of CNCs can provide an efficient methanol barrier and affect proton conductivity [145,146,147].

**Figure 4:**
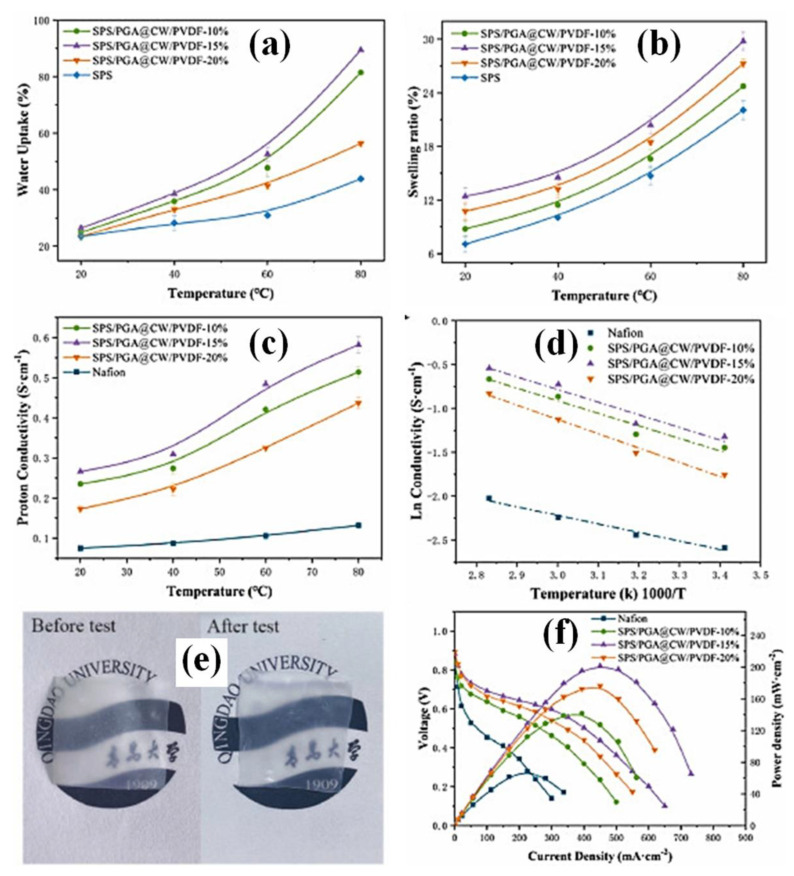
Nafion and different concentrations of SPS–PGA@CW–PVDF (10, 15, and 20%) membranes performances: (**a**) water uptake, (**b**) dimensional stability, (**c**) proton conductivity, (**d**) Arrhenius plots, and (**f**) DMFC unit cell performances (under 65 °C, 100% RH). (**e**) Digital images of SPS–PGA@CW–PVDF before and after water soaking (80 °C for 12 h). Reprinted with permission from Ref. [154]. Copyright © 2022 Elsevier B.V. (License Number: 5442730970358).

Owing to its highly non-reactive thermoplastic fluoropolymer nature, poly(vinylidene fluoride) (PVDF) has been widely utilized as a separator/membrane candidate for fuel cell systems [148,149,150,151,152]. A solution casting process was utilized to develop a CNC combined with a PVDF membrane, and different H_2_SO_4_ concentrations were utilized for hydrolysis treatment [153]. The hybrid membrane prepared with the hydrophobic qualities of PVDF and CNC can provide considerable benefits. This combination has an impact on the membrane morphology and intrinsic properties, and it reduces methanol permeability. The swelling ratio and methanol permeability of CNC-3/PVDF were significantly lower than those of Nafion 117. The determined dimensional stability/swelling ratios at 25 °C (water contact angle) for CNC-3/PVDF and Nafion 117 membranes were 2.22% (70.3°) and 12.28% (104.62°), respectively. Moreover, CNC-3/PVDF attained considerable water uptake (16.41%), IEC (0.84 meq/g), and proton conductivity (7.57 × 10^−2^ mS cm^−1^), whereas the Nafion 117 membrane achieved 8.22% water uptake, 0.84 meq/g IEC and 20.4 × 10^−2^ mS cm^−1^ proton conductivity under similar conditions. The CNC-3/PVDF membrane exhibited excellent selectivity compared to other membranes (CNC-1/PVDF, CNC-2/PVDF, and Nafion 117). The reported selectivity of Nafion 117, CNC-1/PVDF, CNC-2/PVDF, and CNC-3/PVDF are 0.074, 1.170, 1.245, and 28.141 × 10^3^ S.s/cm^3^, respectively. This study provides evidence that CNC/PVDF nanocomposite membranes are potentially attractive PEMs for DMFCs in terms of dimensional stability and methanol permeability [153]. In another report, significant modifications were made to PVDF–cellulose (cellulose whiskers (CW))-containing membranes, including polyglutamic acid (PGA) and sulfonated polysulfone (SPS) [154]. A new type of SPS/PGA@CW-PVDF PEM with heterogeneous dual-interface proton transport channels was prepared using a microimpregnation technique. The composite membranes were developed as follows: (i) CW-PVDF by electrospinning, (ii) carboxylation of CW-PVDF, (iii) PGA@CW-PVDF, and (iv) SPS/PGA@CW-PVDF. As reported in Figure 4a,b, the water absorption and swelling ratios of SPS and SPS/PGA@CW-PVDF gradually increased with an increase in temperature from 20 °C to 80 °C at 100% RH. The water uptake and swelling ratio values of the composite membranes were more significant than those of the pure SPS membrane. Moreover, the increased water uptake and swelling ratio were varied by altering the inclusion of PGA@CW-PVDF (10, 15, and 20%) content in the SPS matrix. Among the different concentrations of SPS, the PGA@CW-PVDF up to 15% revealed an increment in water uptake and swelling ratio. The SPS with 20% of PGA@CW-PVDF membrane revealed the decreased water absorption capability because of the strong interfacial contacts between the nanofiber and SPS in the membrane. However, the presence of functional groups allows water uptake on the SPS-PGA@CW-PVDF-20% membrane [154,155,156,157]. Similarly, SPS-PGA@CW-PVDF membranes resulted in temperature-dependent proton conductivity (20 °C to 80 °C at 100% RH), as represented in Figure 4c. The observed proton conductivities of SPS-PGA@CW-PVDF—10%, 15%, and 20% are dramatically increased compared to those of the Nafion membrane under similar conditions. SPS/PGA@CW-PVDF-15% reached 0.582 S/cm of proton conductivity at 80 °C, which was significantly higher than that of the Nafion membrane. Proton conduction in the SPS-PGA@CW-PVDF membranes was made possible by the interaction of the −COOH and −NH_2_ groups with −SO_3_H in the SPS matrix. Accordingly, the energy barrier for proton migration decreased. Heterogeneous dual interfaces provide enriched proton acceptors and donors that facilitate proton conduction in SPS-PGA@CW-PVDF membranes. Furthermore, the interactions between the functional groups created a complex with the methanol diffusion channels owing to the tortuous structure. This phenomenon increased the mechanical strength of the composite membranes. According to the *E_a_* values of all composite membranes (Figure 4d), the composite membranes tailed both vehicle and Grotthuss processes for proton conduction [154,158]. As shown in Figure 4e, great dimensional stability was observed for the SPS/PGA@CW-PVDF membrane after soaking the membrane in water for 12 h. No size changes were observed for the composite membranes. The DMFC performances (at 65 °C, 100% RH using 5M methanol and oxygen) of the Nafion and different concentrations of SPS/PGA@CW-PVDF membrane polarization curves and power densities are shown in Figure 4f. As compared to the Nafion membrane, the attained maximum power density of SPS/PGA@CW-PVDF-15% was 2.92 times greater. The maximum power density of Nafion and SPS/PGA@CW-PVDF-15% membranes are 68.8 and 201.14 mW cm^−2^, respectively. A high-performance PEM was attained by the presence of PGA@CW-PVDF in the membrane matrix, where PGA@CW-PVDF influenced the generation of heterogeneous dual-interface proton transport channels [154]. 

**Figure 5:**
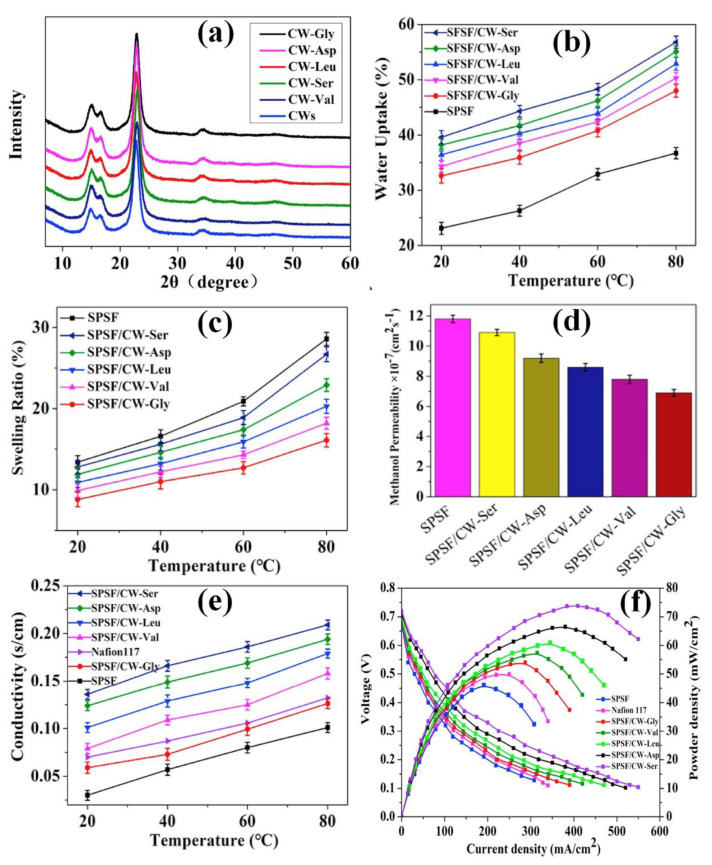
(**a**) XRD spectra of cellulose whiskers (CWs) and CWs functionalized with different amino groups (l–Leucine, l–Asparagine, l–Serine, 5–amino–Valeric acid, and Glycine). (**b**) Water uptake, (**c**) swelling ratio, (**d**) methanol permeability, (**e**) proton conductivity, and (**f**) DMFC single-cell performances (60 °C, 100% RH) of SPSF and different SPSF/CW–AA membranes. Reprinted with permission from Ref. [159]. Copyright © 2018 Elsevier B.V. (License Number: 5442731344740).

A new type of amino-acid-functionalized cellulose whisker (CW) was developed as an efficient PEM for DMFCs [159]. After Fmoc-deprotection, cellulose whiskers were functionalized with amino groups (l-Leucine, l-Asparagine, l-Serine, 5-amino-Valeric acid, and Glycine) by immobilizing the Fmoc-amino acids. By adding amino-acid-functionalized cellulose whiskers (AA-CWs) to sulfonated polysulfone (SPSF with 40% sulfonated degree), an AA-CW incorporated SPSF PEM was developed. According to the XRD spectra of CWs and different AA-CWs (Figure 5a), the crystallinity of the AA-functionalized CWs was similar to that of the CWs. As shown in Figure 5b–e, the incorporation of amino groups (l-Leucine, l-Asparagine, l-Serine, 5-amino-Valeric acid, and Glycine) in the SPSF membrane matrix effectively altered the water uptake, swelling ratio, methanol permeability, and proton conductivity. Compared to the pure SPSF membrane, the presence of different types of AAs in the SPSF membrane increased the water uptake, lowered the swelling ratio, decreased the methanol permeability, and improved the proton conductivity. Owing to the rise and presence of hydrophilic groups (−OH, −SO_3_H, and −NH_2_), the value of WU increased in the modified membrane [159,160,161]. The SPSF/CW-AA membranes exhibited excellent dimensional stability compared to the SPSF membrane because of the uniform dispersion of AA-CWs in the SPSF matrix. Moreover, the interactions between the OH groups on AA-CWs and the SO_3_H groups on SPSF control the swelling of the SPSF membrane after the incorporation of AA-CW in the membrane matrix [159,161]. Compared to the pure SPSF membrane, all the hybrid PEMs exhibited excellent methanol resistance. The generation of a network structure in the membrane matrix by the intermolecular interactions between CWs, AAs, and SPSF in the membrane matrix prevents methanol crossover. The hybrid PEMs with CW-Ser showed an excellent proton conductivity of 0.234 S/cm at 80 °C, which was greater than that of the SPSF and Nafion 117 membranes. In the SPSF/CW-Ser membrane, proton-conducting channels are created by additional amino acids [26]. Considering the functional and physiochemical properties, SPSF/CW-Ser attained high performance during DMFC performers (Figure 5f), where the achieved power densities for the Nafion117, SPSF, and SPSF/CW-Ser membranes were 51.323, 45.344, and 73.757 mW/cm^2^, respectively [159]. A new type of hybrid membrane was developed with a ternary composition of CNC [162]. Here, a ternary membrane was developed using CNCs, chitosan, and PVA (denoted as CNC-CS-PVA) with a smaller amount of glutaraldehyde (GA). The performance was evaluated by the addition of CNCs to different types of hydrolysis (HNO_3_, HCl, and H_2_SO_4_). The proton conductivity of the CNC-CS-PVA membrane increased after protonation, and the incorporation of crystalline CNC nanofillers into the PVA matrix created a tortuous path. Thus, it helped to suppress the methanol permeability from 4.19 × 10^−7^ cm^2^/s (PVA membrane) to 3.12 × 10^−8^ cm^2^/s (CNC-CS-PVA membrane) [162]. The resulting performance values of the modified CNC, MCC, and cellulose whisker-containing proton-exchange membranes for DMFC applications are summarized in Table 1. In summary, modified CNC, MCC, and cellulose whisker-containing membranes exhibited considerable properties for DMFC applications. Moreover, the performance of cellulose-based membranes has been further improved by the addition of various additives and polymers. The intrinsic properties and presence of proton-conducting functional groups in the additional materials improved the performance of cellulose-containing membranes with efficient selectivity (improved proton exchange capacity and lowered methanol crossover) and mechanical properties. 

## 3. Cellulose Nanofibers Containing PEMs for DMFCs

**Figure 6:**
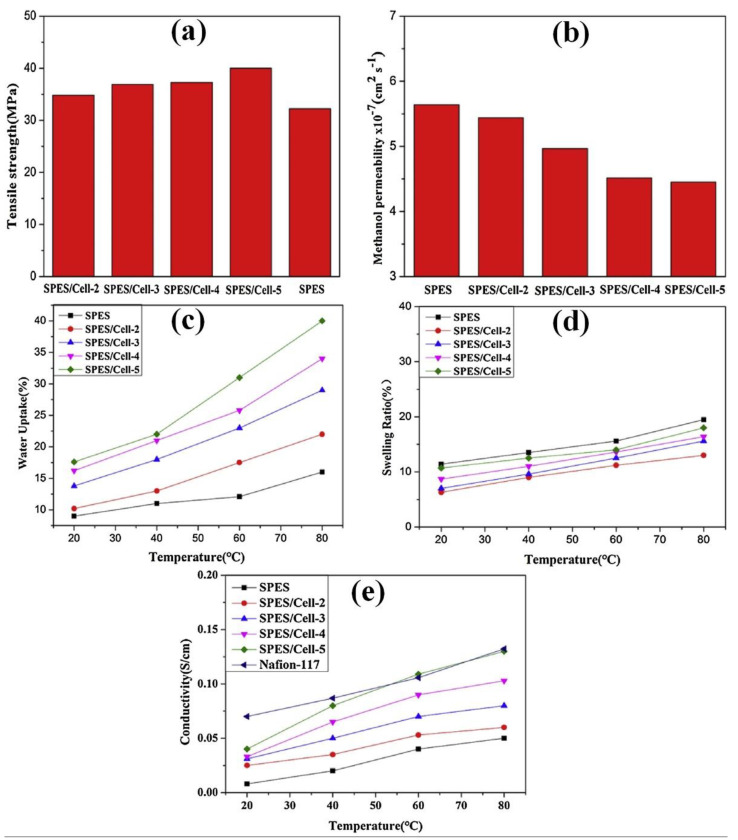
(**a**) Mechanical stability, (**b**) methanol permeability, (**c**) water uptake, (**d**) swelling ratio, and (**e**) proton conductivities of SPES and SPES–CNF membranes. Reprinted with permission from Ref. [170]. Copyright © 2017 Elsevier Ltd. (License Number: 5442740177499).

Cellulose nanofibers (CNFs) are commonly extracted from trees and plants. CNFs were prepared using a dynamic mechanical disintegration process (grinding, microfluidization, and homogenization). In this case, longitudinal nanofibrils were released from the integral microfiber bundles because of the high shear force [163,164]. The fibrils are formed with a cohesive network of H bonds with <100 nm in width and many micrometers in length [163,165,166]. CNFs formed a networked assembly that could serve as a filler or support matrix during composite formation. Additionally, it retains mechanical stability, and the chemical modifiability provided by its hydroxyl groups increases its potential for various applications [167]. Thus, CNFs are considered PEM candidates for DMFCs. Sriruangrungkamol et al. developed nanocellulose (CNF diameter ranging from 18 to 28 nm) membranes by impregnation with different ratios of sulfosuccinic acid (SSA) [168]. The IECs of unmodified and 10.0% *w/v* SSA-modified nanocellulose membranes were 0.005 and 0.069 mmol/g, respectively. Moreover, increased proton conductivity and decreased methanol permeability were attained for the SSA-modified nanocellulose membranes compared to the neat cellulose membrane. Among the different concentrations, the balanced performances of enhanced proton conductivity (0.73 mS cm^−1^) and decreased methanol permeability (1.95 × 10^−6^ cm^2^ s^−1^) were attained for the 5.0% *w/v* SSA sample with CNFs. The presence of SSA with CNF aids cross-linking and the formation of hydrophilic ionic domains, which is the primary reason for the increased performance of CNF membranes [168]. A new type of membrane was developed using CNFs modified with a silica precursor (CNF–Si) and an organosolublefluorine-containing sulfonated polybenzimidazole (s-PBI) copolymer [169]. Here, a bonding agent was used to improve the interfacial interactions between CNF–Si and s-PBI. The inclusion of CNF–Si increased the mechanical characteristics and methanol barrier capability of the s-PBI membranes. The s-PBI/CNF–Si membrane exhibited higher antibacterial activity against Gram-negative bacteria (*Pseudomonas aeruginosa*, *E. coli* O157:H7, and *Escherichia coli*), methicillin-resistant bacteria (*S. aureus*), and Gram-positive bacteria (*Staphylococcus aureus*) [169]. In another approach, Xu et al. impregnated CNF into sulfonated poly(ether sulfone) (SPES) using a solvent casting process [170]. Figure 6a shows the mechanical characteristics of the SPES and SPES–CNF composite membranes. The obtained tensile strengths for SPES composite membranes with a 2%, 3%, 4%, and 5% CNF content were 34.82, 36.9, 37.28, and 40.03 MPa, respectively, whereas the SPES showed lower tensile strength than the composite membranes. The tensile strength of the membrane gradually improved with an increase in the CNF concentration. Similarly, the WU value also increased with the addition of more CNF to the SPES membrane (Figure 6c), where pure SPES showed lower WU at the same temperatures (20 to 80 °C). Numerous −OH functional groups in CNF are well suited for forming hydrogen bonds with water molecules, which effectively influences the WU at high concentrations [170,171]. Moreover, the good interaction between the −SO_3_H functional group in the SPES and the water molecules also increases the water content of the membrane. The presence of absorbed water molecules and the membrane’s −SO_3_H functional group can possibly produce ion cluster formation and proton conductive channels. According to this, the water content behavior of the membrane is also a major factor affecting proton conductivity [170,172]. As shown in Figure 6d, the swelling ratio of the CNF-impregnated SPES membranes was lower than that of pure SPES. The tangled nanofiber network could reduce swelling and improve dimensional stability. Similar to water uptake, the proton conductivity of the membranes (Figure 6e) increased with an increase in the CNF content. Among the different concentrations of CNF in SPES, the maximum value was reached at 5% CNF (0.13 S/cm at 80 °C). The results showed that the proton conductivity was improved up to 1.6 times that of the pure SPES membrane and comparable to that of the commercial Nafion 117 membrane. Additionally, the SPES–CNF composite membranes exhibited considerable benefits in preventing the crossover of methanol through the membrane (Figure 6b). Compared to the pure SPES membrane (5.64 × 10^7^ cm^2^ s^−1^), different ratios of SPES–CNF membranes had reduced methanol permeabilities. Among the different SPES–CNF membranes, SPES–CNF-5 had the lowest methanol permeability (4.45 × 10^−7^ cm^2^ s^−1^). The lowered methanol permeability in the hybrid membrane was due to the more significant nanofiber fraction in the membrane. This proved that the CNFs in the SPES membrane created an internal methanol barrier layer in the overall membrane matrix [170,173].

To further improve the performance of the SPES membrane with CNF, imidazole was incorporated into the CNF (CNF-Im) to enhance the proton transfer mechanism [174]. An acid–base pair-containing membrane was developed using a combination of CNF-Im and SPES. Interestingly, the ion conductivity of SPES/CNF-Im-30 was 0.123 S/cm at 80 °C and 100% RH, which was 2.45 times greater than that of SPES. The formation of long-range proton-conducting channels and additional proton transfer sites (because of the acid–base pair) in SPES/CNF-Im were the primary reasons for the proton conductivity. Moreover, methanol permeability and stability are impacted by the presence of a three-dimensional hydrophilic network structure [174]. In another approach, phosphoric acid (PA) was doped into CNF (CNF-PA) and incorporated into SPES to enhance the overall membrane performance, specifically improving the efficient proton conduction mechanism [175]. An amount of 0.25 mol/L of PA in CNF incorporated into SPES attained the highest conductivity (0.154 S/cm, 80 °C, 100 RH). The enrichment of proton conductivity was attained by increasing the PA doping in the CNF. PA doping provided more proton transport sites to the proton-conducting channels. The efficient incorporation of CNF-PA into the SPES matrix membrane generated a methanol-resistant layer in the membrane, which lowered the permeability of methanol and increased its diffusion resistance [175,176].

**Figure 7:**
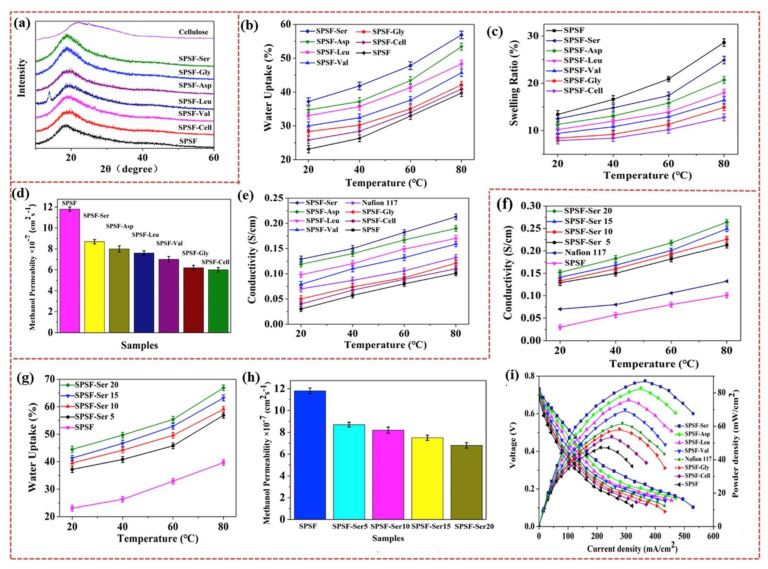
(**a**) XRD spectra, (**b**) water uptake, (**c**) swelling ratio, (**d**) methanol permeability, and (**e**) proton-conductivity of SPSF, SPSF/CNF, and SPSF/CNF–AA membranes. Performances of membranes with different ratios of SPSF/CNF–AA(Ser): (**f**) proton conductivity, (**g**) water absorption, and (**h**) methanol permeability. (**i**) DMFC performances of Nafion 117, SPSF, SPSF/CNF, and SPSF/CNF–AA membranes. Reprinted with permission from Ref. [180]. Copyright © 2019 Elsevier B.V. (License Number: 5442740398615).

Sulfonated polysulfone (SPSF) polymers have been effectively considered efficient membrane candidates for DMFC applications [177,178,179]. According to Zhao et al., the SPSF membrane performance can be further tuned by the CNFs and immobilization of different amino acid (AA) molecules in the CNF structure [180]. The CNFs were functionalized with different AA groups (l-Leucine, l-Asparagine, l-Serine, 5-amino-Valeric acid, and Glycine) and incorporated into the SPSF matrix to develop the hybrid membrane. According to the XRD spectra (Figure 7a), CNF promoted the compatibility and crystallization properties of SPSF. Figure 7b–e illustrate the water absorption, swelling ratio, methanol permeability, and proton conductivity of SPSF, SPSF/CNF, and SPSF/CNF-AA, respectively. Compared to the SPSF membrane, the SPSF/CNF-AA membrane provided higher water uptake, excellent dimensional stability, and effectively controlled methanol crossover. In the SPSF/CNF-AA membrane, water uptake may be significantly enhanced by the presence of functional groups in the CNF and the existence of a three-dimensional network cluster structure of AA. This phenomenon further influences swelling behavior, which is the reason for the increased dimensional stability. In the membrane matrix, the formation of acid–base pairs (between −SO_3_H (SPSF) and −OH/−NH_2_ (CNF-AA)) by the strong electrostatic attractions resulted in the control of excessive swelling in the hybrid membrane SPSF/CNF-AA [180,181]. The lowered methanol permeability of the SPSF/CNF-AA membrane possibly occurred for two reasons. The inclusion of CNFs produces a curved network that possibly lowers the crossover of methanol [173,180]. Additionally, the hydrogen bond formation between the −SO_3_H (SPSF) and −OH/−NH_2_ (CNF-AA) functional groups limits methanol permeability. As compared to pure SPSF, Nafion 117, and other SPSF/CNF-AA membranes, the SPSF/CNF-AA(Ser) membrane showed considerable proton conductivity in all the measured temperature ranges (20 to 80 °C) (Figure 7e). The proton conductivities of the Nafion, pure SPSF, and SPSF/CNF-AA(Ser) membranes were 0.101, 0.132, and 0.213 S/cm, respectively, at 80 °C. The enhanced proton conductivity in SPSF/CNF-AA(Ser) may be attained by the presence of functional groups (−SO_3_H (SPSF), −OH (CNF), and −NH_2_ (AA)) in the membrane matrix, more sites for water to form hydrogen bonds, high water retention in the membrane, acid–base pairs, and the formation of proton channels to transfer protons [124,158,180,182,183]. To further understand the impact of CNF-AA(Ser) on the SPSF membrane, different loading amounts of CNF-AA(Ser) (5, 10, 15, and 20%) were incorporated. The proton conductivity, water uptake, and methanol permeability of the SPSF/CNF-AA(Ser) membranes are shown in Figure 7f–h, respectively. At 80 °C, the SPSF/CNF-AA(Ser) membrane with 20% obtained a proton conductivity of 0.264 S/cm, which was higher than the other SPSF/CNF-AA(Ser) concentrations. In the SPSF/CNF-AA(Ser) membrane, CNF-AA(Ser) was uniformly dispersed in the SPSF matrix. The proton transfer channels in SPSF/CNF-AA (Ser) were effectively increased by the CNF-AA(Ser) content in the membrane matrix. Similar benefits were attained for methanol permeability, where methanol permeability decreased with increasing CNF-AA(Ser) in SPSF/CNF-AA(Ser). Based on proton conductivity and methanol permeability, the SPSF/CNF-AA(Ser) membrane exhibited excellent DMFC performance compared to SPSF, SPSF–CNF, Nafion 117, and other SPSF/CNF-AA membranes (Figure 7i) [180]. 

**Figure 8:**
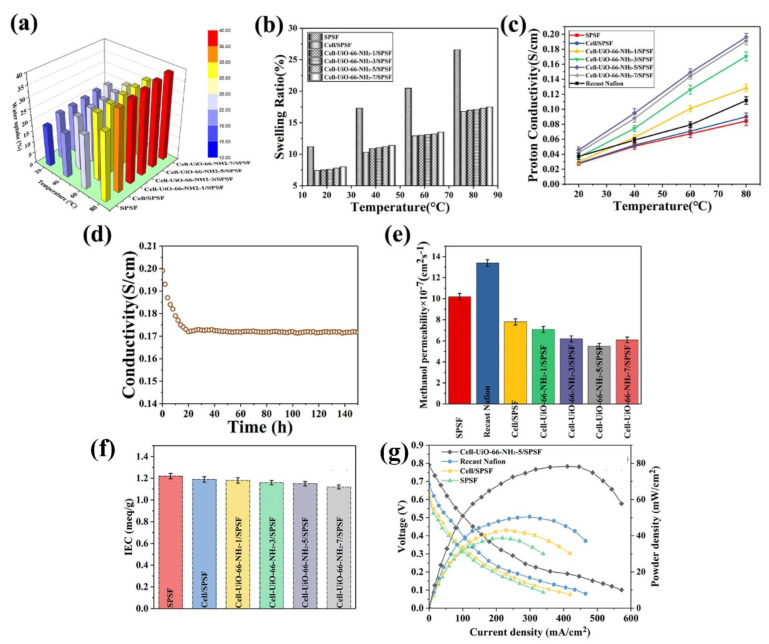
(**a**) Water uptake, (**b**) swelling ratio, (**c**) proton conductivity of SPSF and SPSF/CNF–UiO–66–NH_2_ (0 to 5 wt%). (**d**) Time-dependent proton conduction of SPSF/CNF–UiO–66–NH_2_ (5 wt%) membrane (at 80 °C, 100% RH). (**e**) Methanol permeability and (**f**) IEC of SPSF and SPSF/CNF–UiO–66–NH_2_ membranes. (**g**) DMFC performance with SPSF, SPSF/CNF, and SPSF/CNF–UiO–66–NH_2_ membranes. Reprinted with permission from Ref. [187]. Copyright © 2021 Hydrogen Energy Publications LLC. Published by Elsevier Ltd. (License Number: 5442740604563).

Metal–organic framework (MOF) incorporated membranes have recently been shown to enhance DMFC performances [184,185,186]. Similarly, an investigation proved that MOF-functionalized (UiO-66-NH_2_)-CNF improved the intrinsic properties of the SPSF membrane for DMFC applications [187]. The water uptake and dimensional stability of the composite membranes were comparable to those of the pure SPSF membranes (Figure 8a,b). In general, the increased water absorption in the membrane causes significant swelling. However, the SPSF/CNF-UiO-66-NH_2_ membrane displayed a better water absorption performance and a lower swelling ratio. In the composite membrane, the higher water uptake was ascribed to the high concentration of −OH functional groups. The 3D structure of CNFs in the composite membrane was reasoned for efficient dimensional stability. The proton conductivity of all membranes increased with increasing the temperature from 20 to 80 °C, and the proton conductivity was varied for each membrane (Figure 8c). The proton conductivities of the SPSF/CNF-UiO-66-NH_2_ membranes increased with raising the concentration of UiO-66-NH_2_ from 0 to 5 wt% and slightly declined after 5 wt%. The SPSF/CNF-UiO-66-NH_2_ membrane exhibited good proton conductivity (0.196 S cm^−1^ at 80 °C). In hybrid membranes, the presence of different functional groups and the 3D structure of CNF-UiO-66-NH_2_ lead to high proton conductivity and an efficient proton transport channel. Thus, the CNF incorporated with UiO-66-NH_2_ in the SPSF membrane exhibited excellent proton conductivity compared to the pure SPSF, SPSF–CNF, and recast Nafion membranes under similar measurement conditions. However, the proton conductivity gradually decreased to 0.17 S/cm from an initial value of 0.196 S/cm within the first 20 h (Figure 8d). Subsequently, the proton conductivity of the hybrid membranes remained stable. As shown in Figure 8e, the pure SPSF and recast Nafion membranes had far higher methanol permeability than the SPSF/CNF-UiO-66-NH_2_ membranes. The determined methanol permeability SPSF and SPSF/CNF-UiO-66-NH_2_ membranes are 10.2 × 10^−7^ and 5.5 × 10^−7^ cm^2^ s^−1^, respectively. Compared to the pure SPSF membrane, the presence of CNF-UiO-66-NH_2_ in the SPSF membrane blocked methanol crossover and created a longer route. This phenomenon improves the resistance to methanol diffusion, and additional methanol molecules can be trapped by UiO-66-NH_2_’s porous shape [176,187,188]. A low IEC was observed for the composite membrane (Figure 8f). The interaction between CNF-UiO-66-NH_2_ and SPSF limits the dissociation of protons from the acidic groups [158,187]. Thus, the SPSF/CNF-UiO-66-NH_2_ membrane had a lower IEC than that of the pure SPSF membrane. The DMFC unit cell constructed using the SPSF/CNF-UiO-66-NH_2_ membranes demonstrated a high power density (78 mW/cm^2^). The unit cell performance of SPSF/CNF-UiO-66-NH_2_ is 101% greater than the SPSF membrane (Figure 8g) [187]. 

## 4. Cellulose Acetate-Containing Proton-Exchange Membranes for DMFCs

Cellulose acetate, a semi-synthetic polymer, is a chemically modified cellulose biopolymer. Cellulose acetate has been developed by the acetylation of hydroxyl groups in cellulose [189,190,191,192]. The hydrophilicity of the cellulose structure is reduced when more hydroxyls are replaced with acetyls [167,193]. Eldin et al. developed a sulfonated cellulose acetate (SCA) membrane as a proton-exchange membrane for DMFCs [194]. Here, epichlorohydrin (ECH) was used to activate cellulose acetate, and the membranes were then doped with a sodium sulfite solution. Compared to 0.9 meq/g for Nafion^®^ 117, the IEC was expected to be between 0.369 and 0.996 meq/g. Additionally, Fenton’s reagent showed that the membrane had a long lifespan. Lower uptake of methanol, reasonable dimensional stability, and good mechanical characteristics (49.25N) were attained by the membrane. Under identical circumstances, the SCA membrane’s methanol permeability was much lower than that of Nafion^®^ 117. These findings indicate that low-cost SCA membranes are suitable polyelectrolytes for DMFCs [194]. Similarly, a new kind of polyelectrolyte membrane of phosphorylated cellulose acetate membrane (PCA) was developed using epichlorohydrin [195]. According to the orthophosphoric acid concentrations between 0.25 and 2M, the IEC of the PCA membranes varied between 0.4 and 2 meq/g. The PCA membrane showed lower and higher methanol and water uptake, respectively. The PCA membrane methanol uptake decreased to 4.0723% from 9.0225% compared to as-received CA membranes. Thus, the methanol permeability of the PCA membrane is drastically decreased than that of the CCA and Nafion membranes. The methanol permeability of PCA and Nafion membranes are 2.4 × 10^−15^ cm^2^/s and 1.14 × 10^−9^ cm^2^/s, respectively. The affordability of the CA polymer and its simplicity in manufacturing make it a promising option [195]. For DMFC, aminated proton-exchange membranes based on CA were developed using ECH and ethylene diamine (EDA). The aminated CA-based membranes showed outstanding dimensional stability. Moreover, the methanol permeability (4.54 × 10^−17^ cm^2^/s) is lower than that of the Nafion 117 membrane. Additionally, varying the EDA contents in the membrane altered intrinsic properties and exhibited a significant improvement [196].

Khalifa et al. successfully used a solution-casting method to fabricate Ph-CA/TiO_2_ (phosphorous-functionalized) nanocomposite membranes using TiO_2_ NPs [197]. The IEC of the Ph-CA membrane was 0.6 and 0.81 meq/g at 25 and 80 °C, respectively. However, the IECs of 5 wt% TiO_2_ incorporated into Ph-CA membranes were significantly altered to 1.13 and 2.01 meq/g under similar conditions. According to AFM of the pure Ph-CA membrane, bright and dark phases are assigned to the hydrophobic polymer matrix and the hydrophilic phosphonate groups, which confirms the presence of hydrophobic and hydrophilic characteristics in the membrane [197,198]. In the Ph-CA-5/TiO_2_ nanocomposite membrane, the surface morphology confirmed the efficient dispersion of TiO_2_ nanoparticles in the membrane. The addition of TiO_2_ influenced the mechanical strength of the Ph-CA membrane, where the mechanical stability gradually increased by adding TiO_2_ content from 0 to 7.5 wt%. The mechanical stabilities of the Nafion, Ph-CA, and Ph-CA/TiO_2_-7.5 wt% were 37.7, 18.2, and 58 MPa, respectively. Additionally, the tensile strength of the membranes noticeably decreased when the TiO_2_ content increased to 10 wt%. In higher concentrations, the agglomeration of TiO_2_ nanoparticles in the membrane lowers the mechanical stability [197]. The methanol permeability of the nanocomposite membrane varied by altering the TiO_2_ concentrations in the membrane. As compared to the Ph-CA membrane (2.27 × 10^−16^ cm^2^/s), the methanol permeability is dropped for Ph-CA/TiO_2_-2.5 wt% (1.25 × 10^−16^), Ph-CA/TiO_2_-5 wt% (0.98 × 10^−16^ cm^2^/s), and Ph-CA/TiO_2_-7.5 wt% (2.1 × 10^−16^ cm^2^/s), respectively. It indicates that the Ph-CA membrane with TiO_2_ limits the permeation of methanol crossover across the membrane. Additionally, this composite membrane may prohibit the poisoning of the cathode catalyst. The higher amount of TiO_2_ (10 wt%) in the Ph-CA exhibited higher methanol permeability (3.5 × 10^−16^ cm^2^/s) than the pure Ph-CA and other Ph-CA/TiO_2_ membranes. Jiang et al. identified a similar tendency [197,199]. In the as-prepared Ph-CA membranes, the higher methanol diffusion through the membranes occurred with an increase in interlayer spacing because of the holey-phosphonate structure. In Ph-CA/TiO_2_-2.5 wt% and Ph-CA/TiO_2_-5 wt% membranes, the excellent amount and dispersion of hydrophilic TiO_2_ lead to prohibiting the methanol crossover through the membrane. Therefore, it is probable that the efficient amount of inorganic additives with modified CA can serve as excellent PEM for DMFC [197]. Polymerized triazole (PTZA) and polyacrylic acid (PAA) copolymer were used as reinforcement to create a new kind of PEM based on cellulose triacetate (CTA) [200]. Compared to a pure CTA membrane, the CTA/PTZA-20 membrane has greater proton conductivities (2.313 × 10^−4^ S/cm) and lower methanol permeability (1.773 × 10^−7^ cm^2^/s). The triazole moiety aids in increasing proton conduction and works as a proton facilitator. Thus, the CTA/PTZA-20 membrane had a higher proton conductivity than the CTA membrane. In addition, the hydrolytic stability and oxidative tolerability of the CTA membrane are considerably increased after developing the membrane with PTZA-20 as CTA/PTZA-20, where they were increased by 97.78% and 99.6%, respectively, for the CTA/PTZA-20 membrane. According to the enhanced performances of PTZA copolymer-reinforced CTA membrane, it can be considered as PEM for DMFC applications [200]. The resulting performance values of CNF and cellulose acetate-containing proton-exchange membranes for DMFCs are summarized in Table 2. 

## 5. Conclusions

DMFCs have been extensively developed as an alternative power source for application in portable electronic devices and transportation fields due to their low cost, high efficiency, high durability, and environmental friendliness using methanol as a liquid fuel. The membrane in DMFCs plays a significant role during its operation. A membrane with the required characteristics, such as low methanol crossover, high ionic conductivity, and high chemical and mechanical stability, results in high DMFC performance. The drawbacks of high cost, high methanol crossover, and operating temperature of commercial Nafion membranes limit their DMFC applications. Thus, various biopolymers have gained increased scientific attention owing to their low cost, hydrophilicity, renewability, and biodegradability. In this review, we summarize cellulose and cellulose derivatives as PEMs for the development of biopolymer-based electrolytes for DMFCs. Cellulose has gained positive attention in PEM development because of its low cost, high hydrophilicity, renewability, easy chemical modification, and enhanced thermal and mechanical stability attributed to its notable DMFC performance. This review elucidates the role of cellulose modified by grafting, cross-linking, acid doping, and composites with different polymers (fluorocarbon and hydrocarbon) and inorganic materials as PEMs in DMFC performance. The hydrophilic nature of cellulose in polymer composite membranes enhances the interfacial interaction with the polymer, thereby enhancing the tensile strength of the membrane. Additionally, membranes with high cellulose content exhibited higher hydrophilicity. The cellulose composite membrane, developed by grafting and cross-linking, exhibited higher chemical and mechanical stability than the membranes developed through doping methods. The nano-scale cellulose (NC) in the composite membrane formed a dense network and channel, thus exhibiting lower methanol crossover. Composite membranes developed using cellulose or its derivatives as enhancers rather than the main matrix exhibit excellent mechanical and ion-conducting properties equal to or higher than those of commercial PEMs. However, composite membranes with cellulose or its derivatives in bulk increase the membrane hydrophilicity, resulting in excessive water uptake and membrane swelling. Prospects rely on cellulose membrane modification by filling with proper swelling inhibitor molecules and alteration in the ion transport pathway, providing solutions for cellulose as the primary matrix in PEMs. Alterations in cellulose materials by phosphorylation, sulfonating, chemical cross-linking, and filling with inorganic particles have been performed to enhance their mechanical properties and ionic conductivities. Moreover, the methanol permeability can be controlled by modifying the cellulose structure and its functional properties. This phenomenon can be further tuned by generating a long path for the moment of methanol species through the membrane or blocking by the excellent cross-linking and additives in the overall membrane. To reduce the cost, area, and time required for cellulose extraction, the microbial mode of extraction was selected for its positive benefits. Here, microbes have utilized agricultural and industrial wastes as a nutritional source for the commercial production of cellulose, with a small area and a very short period. These types of cellulose exhibit a higher mechanical strength than plant-derived cellulose. In the future, cellulose-based membranes will be used as PEMs in DMFCs with enduring scientific and technological improvements.

## Figures and Tables

**Table 1:** Physicochemical, methanol permeability, and DMFC performances of different types of cellulose-containing membranes.

Cellulose/Cellulose Derivatives	Functional Group/Polymers/Additives/Dopants	IEC (meq/g)	Water Uptake (WU) and Swelling Ratio (SR)				Proton Conductivity (IC)		Methanol Permeability cm^2^/s	Fuel Cell Test (mW cm^−2^)	Ref.
			T (°C)	WU (%)	MU (%)	SR (%)	T (°C)	IC (S/cm)			
	Nafion 117	0.3450							2.09 × 10^−6^	34.95 (at 80 °C)	[130]
CNC	CMP1-DAC	0.0344							2.91 × 10^−7^	16.21	
CNC	CMP2-DAC	0.0800							2.46 × 10^−7^	25.48	
CNC	CMP3-DAC	0.0629							2.66 × 10^−7^	28.62	
CNC	TAU1-DAC	0.0955							3.39 × 10^−7^	26.19	
CNC	TAU2-DAC	0.1553							2.96 × 10^−7^	30.60	
CNC	TAU3-DAC	0.1667					100	0.1528	3.89 × 10^−7^	34.0	
CNC	Cys1-DAC	0.0408							2.57 × 10^−7^	20.70	
CNC	Cys2-DAC	0.0518							2.87 × 10^−7^	25.76)	
CNC	Cys3-DAC	0.0519							3.01 × 10^−7^	27.68 (80 °C)	
CNC							100	0.019			[133]
CNC	2,6-diaminopurine						100	0.222	1.41 × 10^−7^		
sulfated cellulose	25% GA—3 h						25	3.3 × 10^−5^			[134]
sulfated cellulose	15% GA—12 h						25	1.7 × 10^−5^			
sulfated cellulose	15% GA—3 h						25	3.7 × 10^−5^			
sulfated cellulose	25% GA—12 h						25	0.69 × 10^−5^	8.28 × 10^−9^		
MCC		0.137		22.52	5.83			0.073 × 10^−3^	8.85 × 10^−7^		[141]
MCC	Im	0.298		38.68	1.87			0.214 × 10^−3^	4.42 × 10^−7^		
MCC	PTA	0.359		36.66	3.77			0.106 × 10^−3^	3.54 × 10^−7^		
	Nafion 117	0.86						0.980 × 10^−3^	12.3 × 10^−7^		
CNC		0.463		47.22	15.94			5.32 mS/cm	9.49 × 10^−7^		[139]
CNC	PTA	2.367		50.68	7.55			13.17 × 10^−3^	8.28 × 10^−7^		
CNC	Im	1.253		58.54	5.38			14.98 × 10^−3^	7.49 × 10^−7^		
CNC	PTA-Im	1.972		75.61	3.82			6.34 × 10^−3^	6.29 × 10^−7^		
	Nafion 117	0.890		20.52				31.60 × 10^−3^	5.65 × 10^−5^		
CNC				36.6	15.9			1.88 ± 0.10 × 10^−3^	7.75 ± 1.28 × 10^−6^		[140]
CNC	m-PTA							7.29 ± 0.29 × 10^−3^	6.28 ± 2.13 × 10^−6^		
CNC	Im	1.253						3.37 ± 1.86 × 10^−3^	6.76 ± 2.17 × 10−6		
CNC	Im/m-PTA-1	1.450						19.09 ± 0.16 × 10^−3^	4.85 ± 1.96 × 10^−6^		
CNC	Im/m-PTA-3	1.578						20.91 ± 0.66 × 10^−3^	4.13 ± 1.26 × 10^−6^		
CNC	Im/m-PTA-5	1.885		50.68	3.19			31.88 ± 0.31 × 10^−3^	1.74 ± 1.48 × 10^−6^		
	Multilayer membrane						70	100.79 × 10^−3^		65.02	[145]
	NR211/NR212/ NR212						70	41.2 × 10^−3^		66.48	
CNC	NR211/CNC/ NR212/NR212							-		39.96	
	NR211/PTFE/ NR212/NR212						70	62.71 × 10^−3^		75.53	
CNC	/NR211/NR212/ CNC/212/212/211/CNC/212/212						70	84.25 × 10^−3^		54.73	
	Nafion 115						70	100.79 × 10^−3^		65.02	
CNC-1	PVDF	0.25	25	5.56	14.28			0.502 × 10^−5^	4.29 × 10^−9^		[153]
			50	23.33	17.91						
			80	37.93	23.44						
CNC-2		0.35	25	4.4	20.48			1.07 × 10^−5^	8.59 × 10^−9^		
			50	17.65	23.08						
			80	28.95)	28.99						
CNC-3		0.84	25	2.22	16.41			7.57 × 10^−5^	2.69 × 10^−9^	8.65	
			50	11.43	22.95						
			80	15.19	45.59						
	Nafion 117	0.84	25	12.28	8.22			20.4 × 10^−5^	2.74 × 10^−6^	19	
			50	13.53	14.64						
			80	18.25	18.10						
Cellulose whiskers (CW)											[154]
CW/PVDF-10%	SPS/PGA			81.450		24.73			2.34 × 10^−7^		
CW/PVDF-15%	SPS/PGA			89.407		29.77	80	0.582	2.05 × 10^−7^	201.14 (65 °C 100% RH)	
CW/PVDF-20%	SPS/PGA			56.380		27.29			4.59 × 10^−7^	-	
	SPS			43.878		22.07			-	-	
	Nafion								14.51 × 10^−7^	68.8	
	SPSF								11.8 × 10^−7^	45.344	[159]
CW-Ser 10%							80	0.234	7.6 × 10^−7^	73.757	
	Nafion 117								-	51.323	
	PVA							3.58 × 10^−5^	4.19 × 10^−7^		[162]
CNC	PVA and CS							5.92 × 10^−4^	3.12 × 10^−8^		
	Nafion 117							8.45 × 10^−3^	2.07 × 10^−6^		

**Table 2:** Different kinds of cellulose-containing membranes for DMFC applications—physicochemical and unit cell performances.

Cellulose/Cellulose Derivatives	Functional Group/Polymers/Additives/dopants	IEC (meq/g)	Water Uptake (WU) and Swelling Ratio (SR)			Proton Conductivity (IC)		Methanol Permeability cm^2^/s	Fuel Cell Test (mW cm^−2^)	Ref.
			T (°C)	WU (%)	SR (%)	T (°C)	IC (S/cm)			
CNF		0.005	RT	50	-	RT	0.45 × 10^−3^	2.24 ± 0.09 × 10^−6^	-	[168]
CNF	0.1%*w/v* SSA	0.0065	RT	37.5	-	RT	0.325 × 10^−3^	0.73 ± 0.12 × 10^−6^	-	
CNF	1%*w/v* SSA	0.01	RT	29	-	RT	0.2 × 10^−3^	0.35 ± 0.12 × 10^−6^	-	
CNF	3%*w/v* SSA	0.034	RT	29	-	RT	0.125 × 10^−3^	1.01 ± 01 × 10^−6^	-	
CNF	5%*w/v* SSA	0.043	RT	50	-	RT	0.75 × 10^−3^	1.96 ± 0.11 × 10^−6^	-	
CNF	10%*w/v* SSA	0.068	RT	60	-	RT	3.17 × 10^−3^	-	-	
	SPES	-	-	-	-	-	-	5.64 × 10^−7^	-	[170]
CNF—2%	SPES	-	-	-	-	-	-	5.44 × 10^−7^	-	
CNF—3%	SPES	-	-	-	-	-	-	4.97 × 10^−7^	-	
CNF—4%	SPES	-	-	-	-	-	-	4.51 × 10^−7^	-	
CNF—5%	SPES	-	-	-	-	80	0.13	4.45 × 10^−7^	-	
	SPES	-	-	-	-	-	-	5.64 × 10^−7^	-	[174]
	Nafion	-	-	-	-	80	0.132	14.6 × 10^−7^	-	
CNF	Im and SPES	-	-	-	-	80	0.123	-	-	
	SPES	1.34	-	-	-	-	-	5.64 ± 0.5 × 10^−7^	-	[175]
CNF	SPES	1.35	-	-	-	-	-	4.45 ± 0.38 × 10^−7^	-	
CNF	PA 0.1 M and SPES	1.38	-	-	-	-	-	5.2 ± 0.35 × 10^−7^	-	
CNF	PA 0.15 M and SPES	1.43	-	-	-	-	-	4.98 ± 0.4 × 10^−7^	-	
CNF	PA 0.2 M and SPES	1.47	-	-	-	-	-	4.5 ± 0.46 × 10^−7^	-	
CNF	PA 0.25 M and SPES	1.52	-	-	-	80	0.154	4.41 ± 0.5 × 10^−7^	-	
	SPSF	1.35 ± 0.02	-	-	-	-	0.101	-	-	[180]
CNF	SPSF	1.31 ± 0.03	-	-	-	-	-	-	-	
CNF	Gly and SPSF	1.30 ± 0.04	-	-	-	-	-	-	-	
CNF	Val and SPSF	1.29 ± 0.02	-	-	-	-	-	-	-	
CNF	Leu and SPSF	1.28 ± 0.05	-	-	-	-	-	-	-	
CNF	Asp and SPSF	1.23 ± 0.03	-	-	-	-	-	-		
CNF	Ser and SPSF	1.27 ± 0.02	-	-	-	-	0.213	-	87.22 (60 °C, 100% RH)	
	SPSF	-			-			10.2 × 10^−7^	-	
CNF	UiO-66-NH_2_ and SPSF	-	80	38.6	17.3	80	0.196	5.5 × 10^−7^	-	[187]
Sulfated cellulose acetate		-			-	-	-	1.729 × 10^−17^	-	
	Nafion 117	0.909			-	-	-	1.14 × 10^−9^	-	[194]
Phosphorylated cellulose acetate		-			-	-	-	2.4 × 10^−15^	-	[195]
Aminated cellulose acetate		-			-	-	-	4.54 × 10^−17^	-	[196]
Phosphorylated cellulose acetate		-	25	22.5	13.05	-	-		-	[197]
		-	80	47.7	13.46	-	-		-	
Phosphorylated cellulose acetate	TiO_2_—2.5%	-	25	24.3	12.81	-	-	1.25 × 10^−16^	-	
		-	80	49.5	13	-	-		-	
Phosphorylated cellulose acetate	TiO_2_—5%	-	25	23.1	11.59	-	-	0.98 × 10^−16^	-	
		-	80	48	12.08	-	-		-	
Phosphorylated cellulose acetate	TiO_2_—7.5%	-	25	22.8	11.07	-	-	2.1 × 10^−16^	-	
		-	80	46.9	11.54	-	-		-	
Phosphorylated cellulose acetate	TiO_2_—10%	-	25	21	10.35	-	-	3.5 × 10^−16^	-	
		-	80	46.54	10.77	-	-		-	
	Nafion 117	1.13	-	-	-	-	-	1.14 × 10^−9^	-	
Cellulose triacetate	PAA and PTZA	-	-	-	-	-	2.313 × 10^−4^	1.773 × 10^−7^	-	[200]

## Data Availability

Not applicable.
